# Integrated transcriptional analysis unveils the dynamics of cellular differentiation in the developing mouse hippocampus

**DOI:** 10.1038/s41598-017-18287-w

**Published:** 2017-12-22

**Authors:** Giovanni Iacono, Marco Benevento, Aline Dubos, Yann Herault, Hans van Bokhoven, Nael Nadif Kasri, Hendrik G. Stunnenberg

**Affiliations:** 1 Radboud University, Department of Molecular Biology, Faculty of Science, 6500 HB Nijmegen, The Netherlands; 2 Department of Cognitive Neuroscience, Radboudumc, 6500 HB Nijmegen The Netherlands; 3 Department of Human Genetics, Radboudumc, 6500 HB Nijmegen The Netherlands; 4 Donders Institute for Brain, Cognition, and Behaviour, Centre for Neuroscience, 6525 AJ Nijmegen, The Netherlands; 5 Institut Clinique de la Souris, Phenomin, Gie Cerbm, 1 rue Laurent Fries, 67404 Illkirch, France

## Abstract

The ability to assign expression patterns to the individual cell types that constitute a tissue is a major challenge. This especially applies to brain, given its plethora of different, functionally interconnected cell types. Here, we derived cell type-specific transcriptome signatures from existing single cell RNA data and integrated these signatures with a newly generated dataset of expression (bulk RNA-Seq) of the postnatal developing mouse hippocampus. This integrated analysis allowed us to provide a comprehensive and unbiased prediction of the differentiation drivers for 11 different hippocampal cell types and describe how the different cell types interact to support crucial developmental stages. Our results provide a reliable resource of predicted differentiation drivers and insights into the multifaceted aspects of the cells in hippocampus during development.

## Introduction

The hippocampus is an allocortical structure belonging to the limbic system and located in the medial temporal lobe. The hippocampus plays a central role in a variety of cognitive functions including formation of new episodic memories and their classification in time^1^, spatial learning and navigation^2^, imagining of fictitious and future experiences^3^, food intake control^4^ and sleep^5^. In rodents, hippocampal ontogenesis starts prenatally, around embryonic day 11 (E11), and is completed in most of its anatomical and functional features around postnatal days 20/30 (P20/P30), when the mature stage is considered to begin^6^.

Genome-wide transcriptional profiling with microarrays showed that the developmental transcriptome of the hippocampus (from E16 to P30) displays striking dynamic changes which correlate with major developmental hallmarks and cellular events, including neurogenesis and differentiation^7^. Furthermore, adult hippocampus was also shown^8^ to be constituted by a large amount of different, specialized cells including at least ten major cell types and more than 40 subtypes. This cellular diversity is achieved thanks to differentiation drivers whose expression is tightly regulated during hippocampus ontogenesis.

To date, a quantitative, comprehensive assessment of the differentiation drivers in the course of hippocampal development is still lacking mostly due to the inability of bulk RNA to assign expression patterns to individual cell types. Recently, integrated *in-silico* analysis of signature of cell types and bulk datasets has proven to efficaciously overcome the aforementioned limitations^9–11^, providing some insight at the cell type level also in bulk transcriptomes. Here, we generated a developmental dataset of the hippocampal RNA-Seq transcriptome of 5 different developmental stages (embryonic forebrain E15, hippocampus P1, P7, P15, P30) and applied a deconvolution approach which exploits existing single-cell RNA (scRNA) data^8^ to infer putative drivers of differentiation for the major cellular types. Our approach was validated by the literature, as we uncovered numerous well-known genes previously shown to be implicated in the differentiation or maturation of neuronal and glial cells. Importantly, we unveiled many new candidate regulators of cell differentiation which constitute a precious resource providing biological insight into cell differentiation of the central nervous system.

## Results

### Distinct temporal patterns underlie specific developmental programs in the hippocampus

**Figure 1:**
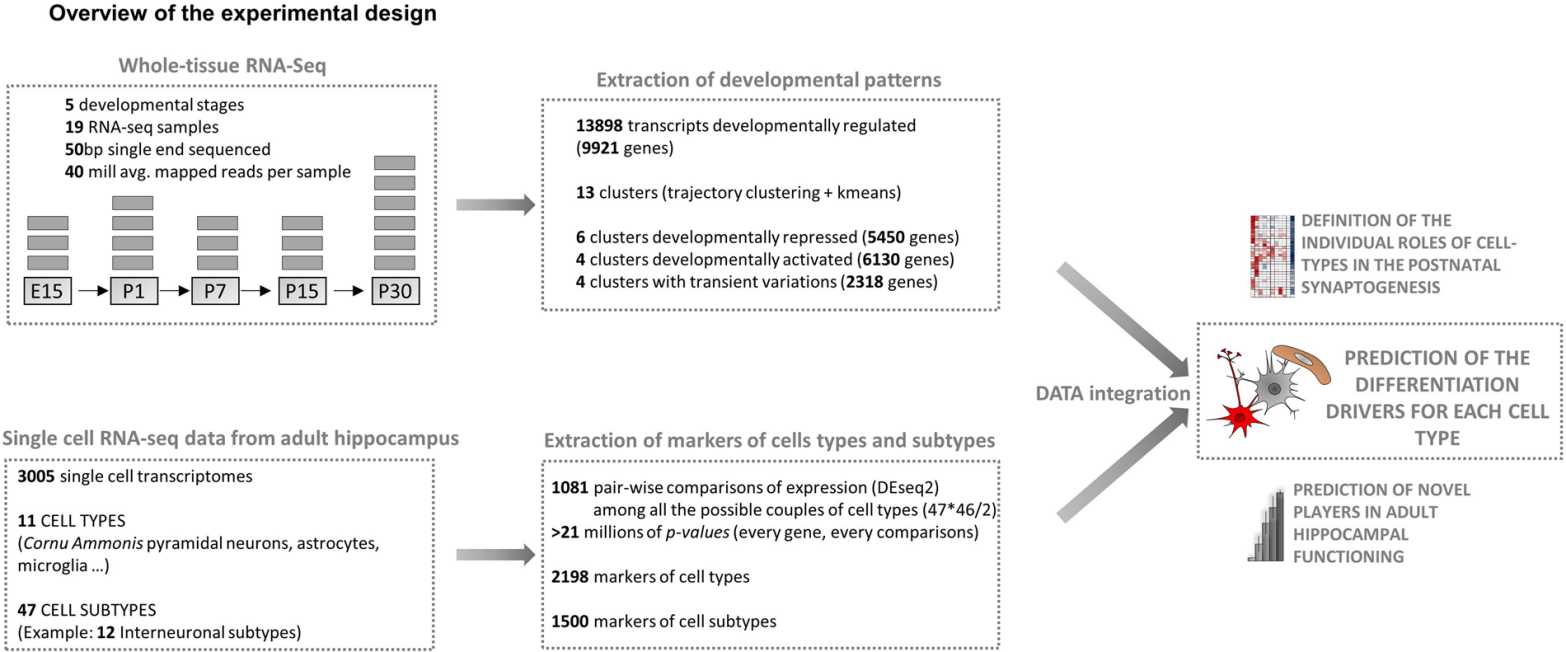
Overview of the experimental design. Schematic representation of the analysis, gray blocks represent the biological replicates (RNA-Seq) for each time point. The newly generated developmental time course of the hippocampal transcriptome is integrated with the single cell data of^8^ to generate an unbiased and fully data-driven prediction of the developmental drivers.

**Figure 2:**
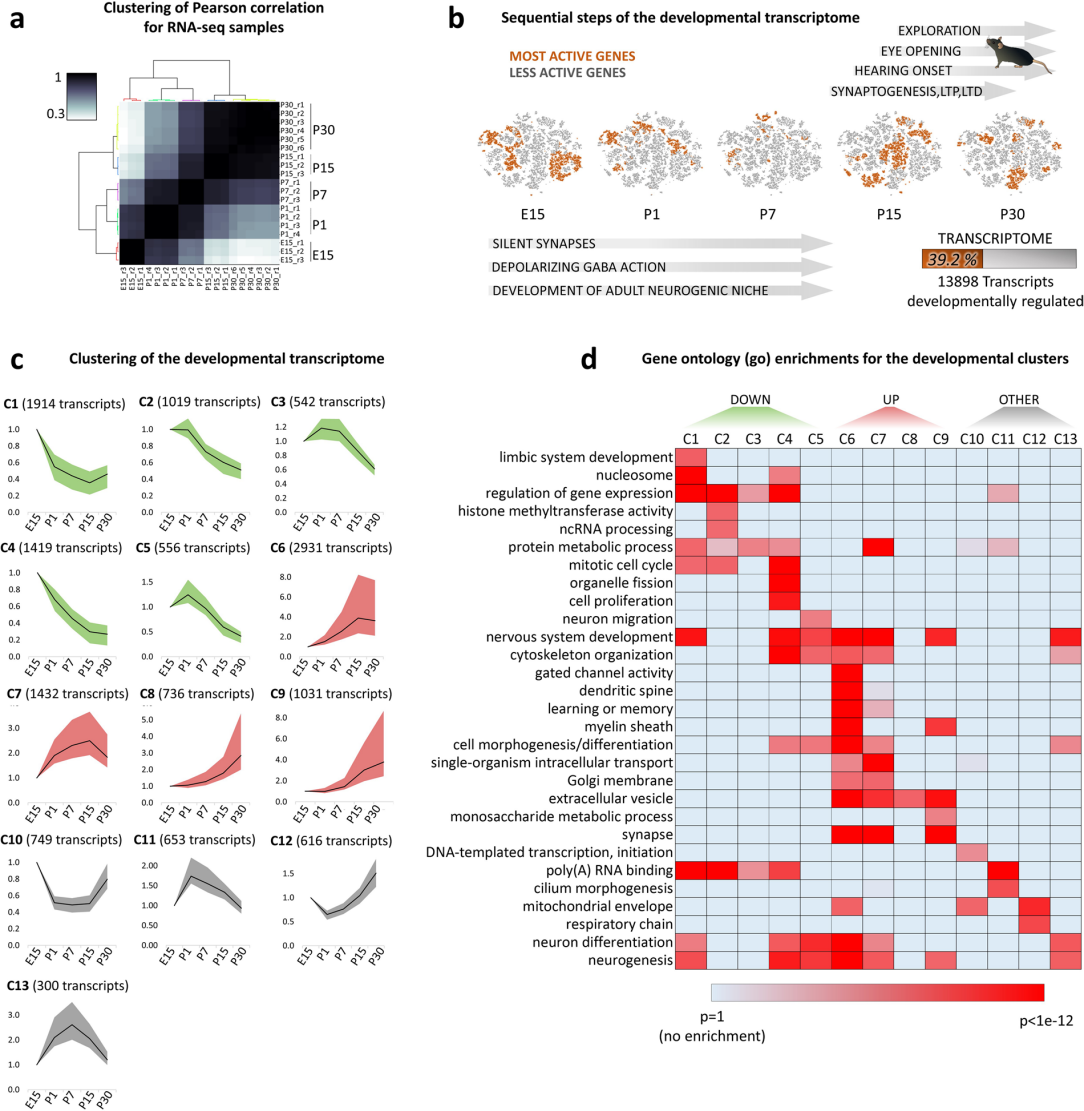
Clustering and functions of the developmental transcriptome. (**a**) Heat-map of Pearson correlations among different RNA-Seq samples. The most representative genes for the developmental phenotype (top 5000 genes) were used for the calculation of correlations (hierarchical clustering with Ward’s linkage). (**b**) Overview of hippocampus development. The major perinatal developmental stages are indicated to the side of the t-sne plots of 13898 transcripts (almost 40% of the transcriptome) found to change expression in the course of hippocampal development. Orange colored genes share the same stage of highest expression and become clustered in the t-sne plots. (**c**) Clustering of the developmental transcriptome (RNA-Seq) in the hippocampus (mean, 25^th^ and 75^th^ percentiles of the expression normalized to E15 stage). (**d**) GO comparisons, heatmap of *Bonferroni* corrected *p-values*.

To characterize the developmental transcriptome of the hippocampus we generated RNA-Seq for the embryonic and postnatal stages E15, P1, P7, P15 and P30. For each stage, at least 3 biological replicates were used (Fig. 1). Analysis of RNA-Seq data identified 13898 transcripts changing expression during perinatal development (DESeq 2 corrected *p-value* < *3e-7*, equal to *Z-score* = 5). Clustering of Pearson correlations of top scoring transcripts, an unbiased method to quantify the degree of similarity between large data sets, shows clear segregation between developmental stages and high concordance of biological replicates (Fig. 2 a), indicating that the measured changes in expression are reliable and reproducible.

Dimensionally-reduced plots (Fig. 2 b) suggest that these 13898 transcripts are regulated in order to be active at specific developmental stages in correlation with the major phenotypic changes observable during development. For this reason, we set out to define the main patterns in gene expression observable throughout hippocampus development. To this end, the transcripts were clustered in 13 different temporal patterns (Fig. 2 c) by means of a multi-step, unsupervised algorithm which firstly estimates the optimal number of clusters and subsequently segregates the genes via k-means clustering (see methods). Of the 13 total clusters, 5 (green color, total of 5450 transcripts) are characterized by a decreasing level of expression throughout the developmental stages, while 4 (red color, 6130 transcripts) are featuring a progressively increase of expression peaking at either P15 or P30. The third class of profiles (gray color, 2318 transcripts) did not show marked differences between initial (embryonic) and final (P30) expression levels but displayed significant transient variations in between.

We next performed gene ontology (GO) analysis to define the putative developmental programs associated with each cluster. Interestingly, an ensemble of several GO terms appears differentially enriched throughout the 13 developmental clusters (Fig. 2 d), suggesting that the division achieved with clustering correlates with a division of biological functions. As previously reported^7^, genes more active in the earlier stages (green clusters) are involved in cellular proliferation (mitotic cell cycle, organelle fission, cell proliferation) or neuronal migration. In contrast, those active in later stages, during synaptogenesis (3th and 4th postnatal week, red clusters), are involved in the establishment of mature neuronal functions (learning and memory, synaptic signaling, others). Notably, even clusters belonging to the same group, as for instance green C1 and C4, or red C6 and C9, appear enriched in markedly different biological functions (Supplementary Fig. S1A,B). The fact that even secondary differences in the patterns give rise to different GO enrichments indicates that each of the 13 clusters corresponds to an informative set of genes.

Intriguingly, we noticed that terms associated with nervous system development and neurogenesis were evident both in developmentally upregulated and downregulated genes. Given the importance of neurogenesis in both ontogeny and disease of hippocampus^12–14^, we sought to use our clustering to investigate the difference between developmental and adult neurogenesis. To this end, we split the genes annotated with the GO term “neurogenesis” (NeuroGenesis Genes, NGGs) in two groups: those expressed at either earlier stages (398 genes, green clusters) or adult stages (445 genes, red clusters). Notably, functional analysis (GO) indicated the two groups of genes to be implicated in distinct pathways (Supplementary Fig. S1C). Specifically, developmental NGGs govern morphological processes through the WNT (*p-value* < 7*.2e-23*) and NOTCH (*p-value* < *1.2e-9*) signaling pathways and are involved in stem-cell development (*p-value* < *3*.7*e-36*) and neuronal differentiation, in particular that of GABAergic neurons (*p-value* < *6.0e-9*), in line with a previous report showing GABAergic neurons to develop earlier than excitatory neurons^15^. Vice versa, adult NGGs appear to regulate synaptic plasticity (*p-value* < *1.7e-54*), associative learning (*p-value* < *3.4e-13*) and neuronal maturation *(p-value* < *4*.2*e-12*) by acting through the ERK1/ERK2 (*p-value* < *1.3e-9*) and RHO (*p-value* < *3.1e-9*) signaling cascades. These results indicate that our dataset and clustering can be effectively used to expose and disentangle the genes and pathways relevant for the hippocampus and more in general brain development. For example, we found that Kv3.2 subunit of Kv3 potassium channels (high frequency channels implicated in epilepsy, Alzheimer’s and spinocerebellar ataxia^16–18^) displays a pattern of developmental expression which diverges from the other three subunits (namely Kv3.1, Kv3.3 and Kv3.4). This indicates that after the 2nd postnatal week the relative abundance of subunits for Kv3 channels is changed (Supplementary Fig. S1D), possibly because of a differential subunit usage. This example serves as a proof of principle that mining our novel results allows to formulate hypotheses which can be used to enhance our mechanistic insights of hippocampal ontogenesis.

### Determining the markers of cell types and subtypes by differential expression analysis

**Figure 3:**
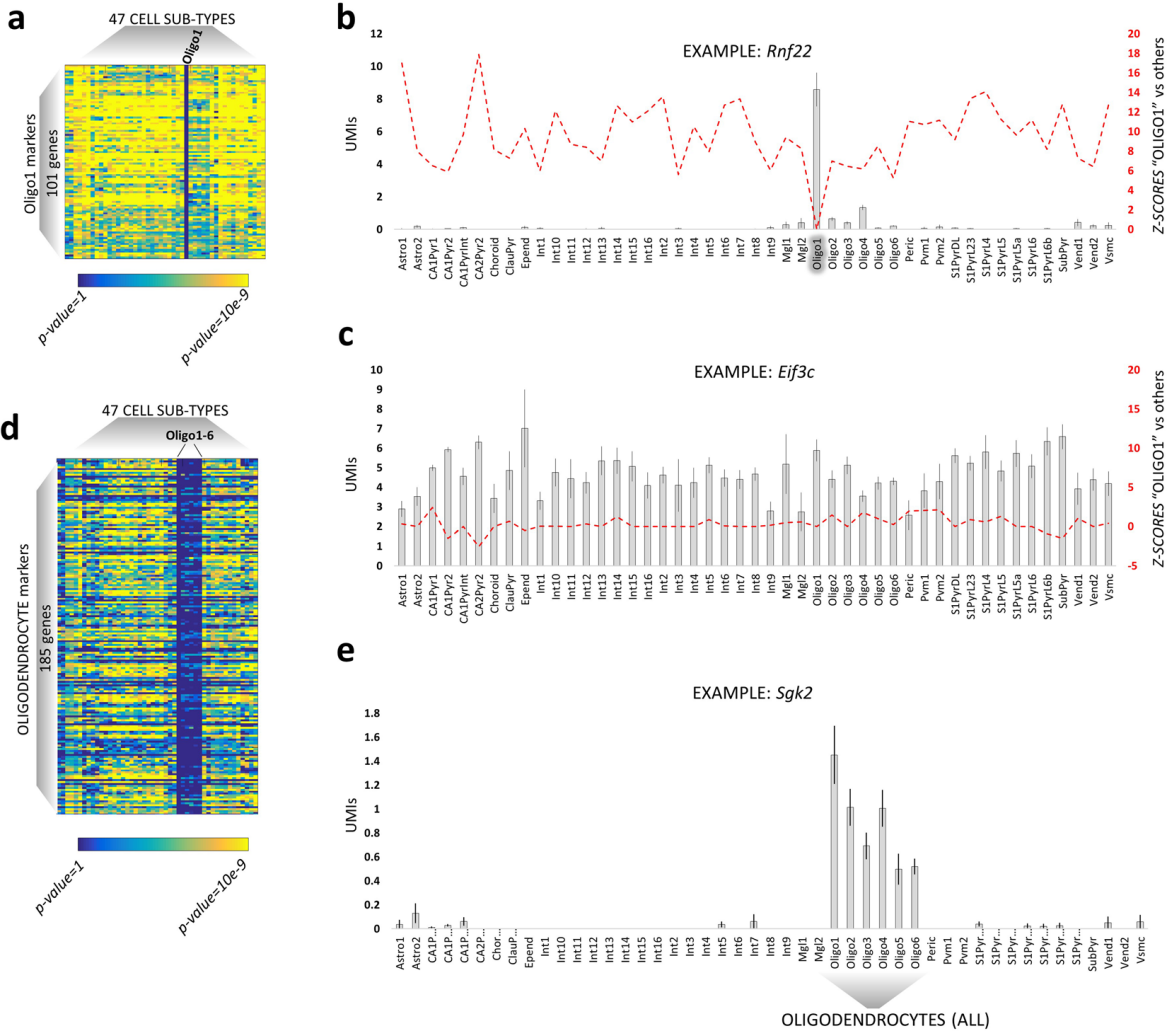
Calculation of markers of cell type and subtypes. (**a**) Heatmap representing the markers of Oligo1, a subtype of oligodendrocytes (immature precursors, as defined in^8^). These markers are significantly more expressed in Oligo1 as they present yellow squares in all the other cell subtype (DESeq 2 corrected *p-values*). (**b**) Histogram (mean +/−SEM, standard error of the mean) of the expression levels (UMIs, unique molecular identifiers, normalized for the library size) of *Rnf22*, a marker of Oligo1. Differential expression analysis (DESeq 2) shows how *Rnf22* expression is significantly higher in Oligo1 as compared to any other cell type (red line, *p-values* in *Z-score* scale, signed to indicate up/down-regulation). (**c**) Non-markers genes, such as *Eif3c*, do not show any significant (red line, *Z-scores* signed to indicate up/down-regulation) change of expression (UMIs, normalized for the library size) throughout the different cell types. (**d**) Heatmap representing oligodendrocytes markers: these 185 genes are expressed in all the 6 oligodendrocytes subtypes whilst silenced in the other cell types (DESeq 2 corrected *p-values*). (**e**) Histogram (mean +/−SEM) of the expression levels (UMIs, normalized for the library size) of *Sgk2*, a marker of all oligodendrocytes.

The hippocampus has a deeply organized structure in which numerous, different cell types and subtypes are functionally or anatomically interconnected and exert their functions in a tightly orchestrated manner. In order to unravel how the different populations of cells collaborate to regulate developmental processes, we set out to isolate the markers of cell types and subtypes by analyzing the scRNA dataset from Zeisel *et al*.^8^. In the latter work, adult cortical and hippocampal cells were classified into 11 types and 47 subtypes (P21-P30 hippocampus, see methods). They also provided lists of markers for every cell type, but not for the cell subtypes. Here, we sought to calculate both the markers of a given cell type and the markers expressed in its subtypes of cells. In this way, for instance, interneuronal markers would not be limited to the genes homogeneously expressed within all interneurons but they will also include the genes expressed in specific interneuronal sub-populations. As a first step, we ran 1081 pairwise comparisons of expression (DESeq 2) among the 47 cellular subtypes. DESeq 2 was shown to be one of the best algorithms to perform differential expression analysis on scRNA data^19,20^. Next, we integrated the resulting statistics in order to extract the markers of each main hippocampal cell type and subtype (see methods). Briefly, the markers of cell subtypes were defined as those genes significantly up-regulated (DESeq 2 corrected *p-values* < 0.05) in one cell subtype compared to each of the others. This method efficiently isolated markers of cell subtypes, as shown for the example of oligodendrocytes (Fig. 3 a–c), whose markers genes, like for instance *Rnf22*, are significantly up-regulated compared to each of the other cell types (yellow colored cells in the heatmap). In addition, genes were considered as markers of a whole cell type (such as interneurons, or oligodendrocytes) when i) they were homogeneously expressed within the cells of a given cell type ii) they were up-regulated in comparison to cells belonging to a different cell type (Fig. 3 d,e). Overall, we found 2198 genes whose expression was significantly higher in one specific cell type compared to the others and 1500 genes that appeared to be expressed only in certain cell subtypes. For example, we identified 156 genes (as *Penk*, or *Fam*4*6a*) which are expressed only in specific interneuronal subtypes (Table S3).

### Integrated analysis of markers and gene ontology (GO) greatly improves the potential of GO

**Figure 4:**
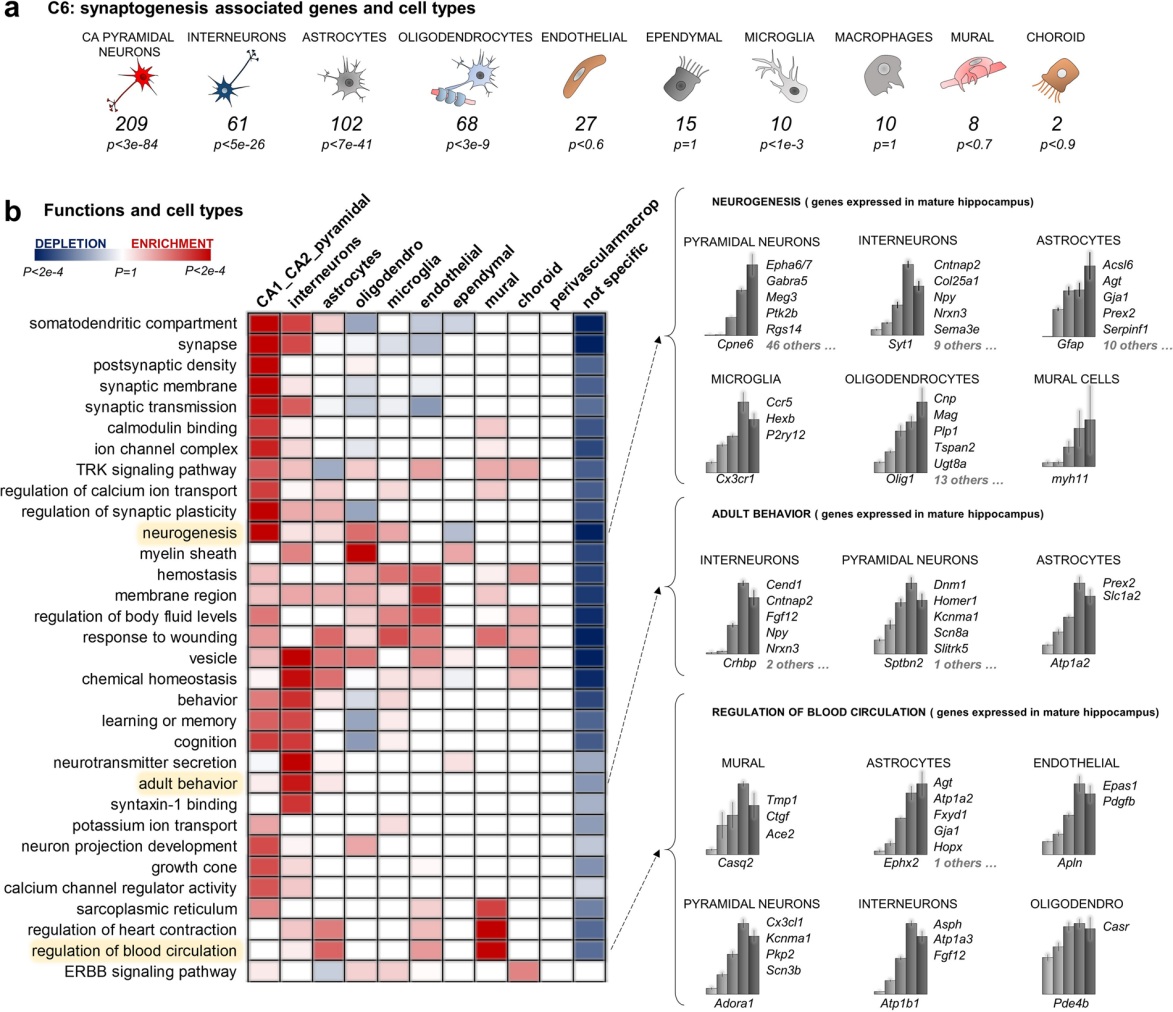
Role of the different cell types in the postnatal synaptogenesis. (**a**) Counts and *Bonferroni* corrected *p-values* of the enrichments of markers in the synaptogenesis cluster C6. *Cornu Ammonis* (CA) pyramidal neurons and *SomatoSensory* (SS) pyramidal neurons are pooled under the label “pyramidal neurons.” (**b**) Identifying the roles of specialized cells in the postnatal synaptogenesis. The heatmap exemplifies the contributions of each of the 11 major hippocampal cell types to the biological functions that were attributed to C6 by standard GO. A red colored cell means that the genes annotated with that certain function (row) mostly belong to that specific cell type (column). Details for some interesting GO terms are shown to the right: lists of cell type markers belonging to C6 and contributing to (adult) neurogenesis, adult behavior and (adult) regulation of blood circulation. Bars represent the expression in the 5 developmental stages (mean +/− SEM).

GO is a widely used tool to gain insights into the functions of gene sets. However, when applied to brain data, GO analysis not always allows for a clear functional interpretation because of the underlying heterogeneity of cell types and hence functions. Here, we set out to improve the potential of GO analysis by integrating it with our lists of markers. To this end, we developed an *in-house* tool based on the cell-type specific enrichment analyses (CSEA^11,21^,) approach to determine the significant GO/cell type interactions. We chose the largest cluster, C6, as a study case. The developmental profile of C6, low at P1 and peaking at P15, indicates that its genes are activated concomitantly with the postnatal synaptogenesis phase. C6 contains almost 3000 transcripts and results enriched in numerous, highly heterogeneous functions (867 GO terms, *Benjamini* corrected *p-value* < 0.01) ranging from “post synaptic density” to “regulation of blood circulation”. Interestingly, C6 is also significantly enriched in the markers of several cell types, from pyramidal neurons to microglia (Fig. 4 a). In order to understand how the functions (GO terms) enriched in C6 are supported by the individual cells types, we used hypergeometric testing with *Bonferroni* corrected *p-values* to quantify the significant GO / cell type interactions (Fig. 4 b). To compute the enrichments, the newly computed lists of markers for cell types, which include also subtypes markers, were used. Interestingly, a significant depletion is detected between most GO terms and the non-marker genes (that is, genes expressed in all cell types), as shown by the blue color of the last column. Vice versa, significant enrichments are detected among GO terms and specialized cell types, as shown by the red squares. This suggests that the main functions of C6 previously determined by GO enrichments appear to be predominantly supported by the specialized cell types.

Notably, the resulting enrichments pinpoint the validity of our newly computed markers, as the functions are correctly associated with the expected cell type. Specifically, we relate neuronal functions (from somatodendritic compartment, *p-value* < *7*.*2e-11*, to neuron projection development, *p-value* < *7*.0*e-3*) to neurons, blood related functions to mural cells (such as regulation of blood circulation, *p-value* < *2*.*5e-4*) or oligodendrocytes functions to oligodendrocytes (myelination, *p-value* < 1.*1e-4*). Furthermore, this computational approach reveals that some of the functions associated with C6 by standard GO enrichment appear to need the support of multiple, heterogeneous cell types. For example, “neurogenesis” is supported by contributions from 6 different cell classes (Fig. 4 b). In contrast, other functions seem to be primarily associated with one single cell type, like “synaptic-transmission” or “cognition”, which are enriched only in pyramidal/inter neurons. Furthermore, our approach also helps to interpret some apparently inappropriate enrichments, like “regulation of heart contraction,” a GO term seemingly extraneous to the hippocampus/brain, which appears to be associated with genes expressed in brain mural cells (vascular smooth muscle cells and pericytes, *p-value* < *4*.*2e-4*) and astrocytes (*p-value* < *0*.037). Moreover, several interesting correlations are uncovered by the heatmap, like a key role of interneurons in the regulation of adult behavior (*p-value* < 6.*9e-4*), supporting the recent literature^22^ linking interneurons to cognitive disorders. In this case, our framework allows to accurately identify the putative interneuronal genes suspected to be implicated in cognitive disorders and expressed concomitantly to the synaptogenesis phase, such as for instance *Npy* or *Crhbp*.

Summarizing, our GO/markers integrated analysis of the 2931 transcripts (1789 genes) activated during the postnatal synaptogenesis and onset of sensory information unveiled an interplay among different specialized cells. The calculated significant matches between cell types and GO terms are in line with evidences in the literature, validating our experimental computation of markers as well as the same GO/markers integrated analysis. This method, which can be implemented in bioinformatics pipelines, allows to exploit previously published signatures of cell types to overcome at least in part the limitation of bulk expression data and gain functional information at the cell type level.

### Exposing postnatal differentiation dynamics by integrating bulk RNA-Seq data and signatures of cell types

**Figure 5:**
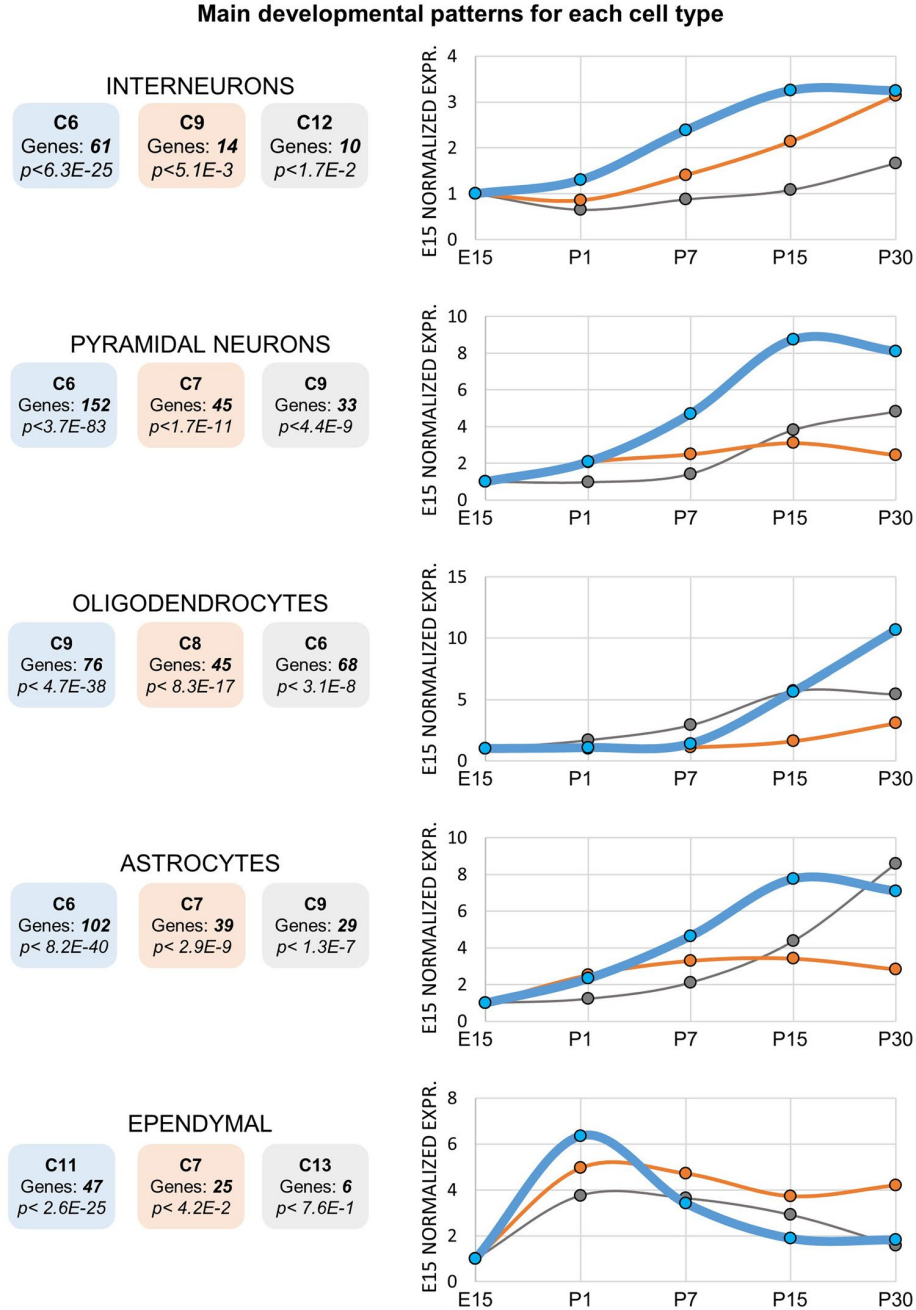
Developmental profiles of hippocampal cell types. Left) The top three enriched developmental clusters (blue 1^st^, orange 2^nd^, gray 3^rd^) are shown for each cell type, alongside the cluster numbers, number of markers and *p-value* of enrichment, *Bonferroni* corrected. Right) The median expression levels of the markers is shown for each cluster (each panel normalized to its own E15 RPKM time point). Blue, orange and gray lines represent the top three enriched clusters.

In the course of hippocampus ontogenesis and maturation, a multitude of specialized cells progressively differentiate, migrate and ultimately form those synaptic and functional connections constituting the adult neural network. However, to date there is no quantitative and comprehensive study defining the abundance of cell (sub)types in the course of hippocampus development. While a deconvolution of absolute proportions of cell types as performed in^23^ is not feasible due to the phenotypic plasticity of differentiating cells, our data still allows to estimate the relative abundance of mature cells. Specifically, here we used our newly established markers of mature cell types with the aim to dynamically estimate the quantity of differentiated, mature cells in the course of neonatal development. To this end, we quantified for each set of markers its enrichments in the previously found developmental patterns (C1-C13). The resulting enrichments (*Bonferroni* corrected *p-values*) are represented in Fig. 5, where for each cell type the top three representative (enriched) developmental patterns are shown.

Overall, mature astrocytes, oligodendrocytes, pyramidal neurons and interneurons become progressively more abundant when approaching the adult-stage (4th postnatal week), as shown by the increasing blue lines. However, while most of the markers of pyramidal neurons, interneurons and astrocytes start increasing already from P1 and reach mature levels at the P15 stage, oligodendrocytes develop only at much later stages, from P15 to at least until P30 stage. This in is line with previous evidences showing that, in P7 rodents, 80% of the white matter is still formed by immature oligodendrocytes^24^.

Interestingly, the markers of each cell type result enriched in multiple developmental patterns. In the astrocytes, for instance, while the majority of the markers reaches mature levels at P15 (102 markers, C6, *p-value* < *8.2e-40*, Fig. 5), another set of astrocyte markers keeps increasing from P15 to P30 (29 markers, C9, *p-value* < *1.3e-7*, Fig. 5). These different astrocytic patterns could be related to the different maturation rates of distinct subtypes of cells. This seems to occur, for instance, in the case of oligodendrocytes, for which the subtype of terminally differentiated oligodendrocytes (as defined in^8^) is enriched (*p-value* < *3.9e-7*, Supplementary Table S4) only in C9 (that is, expression peaking after P15).

Intriguingly, ependymal cells, ciliated glial cells that circulate, absorb and produce cerebrospinal fluid, follow a different trend compared to other cells, as most of their markers decrease during development (after a transient increase in E15 to P1, Fig. 5). This observation is in agreement with the fact that the ependyma covering the hippocampus disappears within 2 or 3 weeks after birth^25^. Also endothelial cells display a significant enrichment in a developmentally repressed pattern (*p-value* < 5*.9e-4*, C5, Supplementary Table 4), in line with the fact that blood vessels are already present during embryonic stages and, after that, they undergo particular forms of plasticity^26^. On the other hand, microglia, mural, choroid cells do not show any significant enrichment in specific developmental patterns (Supplementary Table 4). The low amount of their markers (40, 101, 39 genes respectively) can partially account for this. Lastly, also perivascular macrophages do not present any significant enrichments, suggesting their differentiation dynamics not to be related to the dynamics of the main brain cells types, namely neurons, astrocytes and oligodendrocytes.

Our integrated analysis provided novel quantitative insights into the variegated dynamics of differentiation and maturation of hippocampal cell types, as inferred by the expression patterns of their markers. Apart from oligodendrocytes, which show a slower maturation process, the other major cell types, neurons and astrocytes, reach their adult levels by the end of the 2nd postnatal week. Our results are in agreement with previous biochemical assays, underscoring the validity of our experimental approach.

### Putative regulators of cell-fate commitment, differentiation or maturation

Our data indicates that the majority of neuronal and glial markers display an increasing expression level in the course of development. Those following the opposite pattern, that is repressed in the course of development, intrigued us.

**Figure 6:**
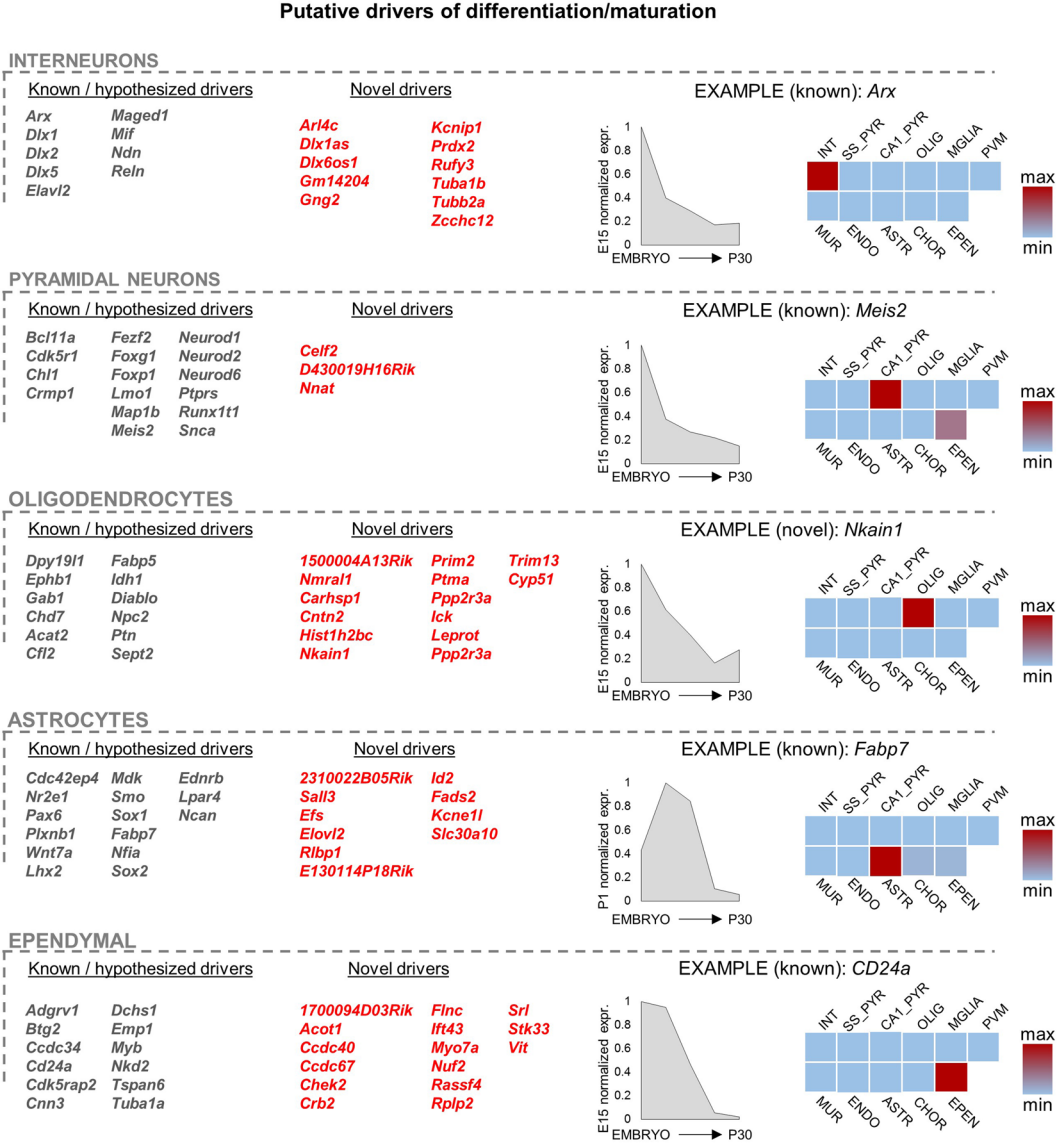
Prediction of the developmental drivers for each cell type. The predicted drivers were selected as those genes i) expressed in only one specific cell type and ii) silenced in the course of development. Predicted drivers which were recognized as previously known or believed ones are colored in gray, while novel ones are colored in red. One example is shown for each cell type: graph representing the developmental expression of the markers (E15, P1, P7, P15 and P30 stages, RPKM normalized to the RPKM of highest expression) and heatmap highlighting the expression levels in the different cell types (P30 data from^8^). The complete lists of predicted drivers can be found in Supplementary Tables S5–S10.

Amongst these divergent markers we could recognize several well-known regulators of neuronal or glial differentiation like *Arx*, *Dlx1* or *Id4* ^27–29^. This led us to hypothesize that those few markers displaying a decreasing expression level during development (which means belonging to green clusters C1-C5) may be directly or indirectly implicated in cell-fate commitment or in differentiation/maturation. Overall, we found 169 of such genes (Fig. 6).

To date, there is no genome-wide, experimentally based database of predicted differentiation drivers like ours, thus we could not validate our results by a direct comparison with other genome-wide datasets. Therefore, we validated our results by means of an extensive literature mining (Supplementary Tables S5–S10) aimed at defining the functions of each of our 169 candidates. Interestingly, several of our candidates turned out to be previously established regulators of cell differentiation/maturation, underscoring the validity of our approach (Fig. 6, gray colored genes). Specifically, for the interneurons, we could single out genes such as *Arx*, *Dlx1*, *Dlx2* or *Dlx5*, all crucial factors in various aspects of differentiation and maturation of interneurons. For pyramidal neurons, we detected amongst others *NeuroD1*, *NeuroD2*, *NeuroD6*, *Meis2*, *Crmp1* and *Fezf2*. For astrocytes we found several well-known differentiation markers, including *Ncan*, *Lpar4*, *Fabp7* and *Nr2e1*. For oligodendrocytes we found, amongst others, *Dpy19l1*, *Ephb1*, *Gab1*, and *Cfl2*. Clearly, not all our candidate markers necessarily drive the differentiation of the cells in which they are expressed, as secreted proteins like *Ptn* (*Pleiotrophin*, a growth factor) are most likely implicated in the development of cell types other than those secreting the factor.

Interestingly, amongst oligodendrocytes, astrocytes and ependymal candidate driver genes we find numerous genes that were previously associated with glial tumors (for instance *Adgrg1*, *Cdc42ep4*, *Mdk*, *Pou3f2*, *Sox21*, *Idh1* or *Cfl2*, supplementary tables), which suggests that their misexpression in cancer reverts the cells to an earlier developmental and proliferative stage.

Overall, the abundance of previously known factors of neurogenesis and gliogenesis validates our approach. Most importantly, our approach uncovered several novel genes that are potentially involved in differentiation or maturation of neuronal and glial cells. Specifically, our integrated analysis predicted 11 novel differentiation drivers for interneurons, 3 for pyramidal neurons, 21 for oligodendrocytes and 10 for astrocytes (Fig. 6, red colored genes). Some of these predicted drivers are transcripts without any functional annotation or information, such as the case of *15*0*0004A13Rik* (oligodendrocytes), *E130114P18Rik* (astrocytes) or *D430019H16Rik* (pyramidal neurons). Others have been poorly studied in the context of CNS, such as for instance the genes *Carhsp1*, a serine phosphoprotein possibly involved in signal transduction, or *Prim2*, a DNA primase exerting a key role in the replication of DNA, both putative oligodendrocyte drivers. Other novel candidates, as well as the results of perivascular macrophages, ependymal cells and mural cells, are discussed in the supplementary notes.

## Discussion

The major limitation of bulk gene expression approaches is the inability to discern the contributions of the different cell types. While in the case of sufficiently homogeneous cell cultures this is not a detrimental problem, in the case of brain regions such as hippocampus it becomes a severe complication^8,30^. Here, we devised a series of bioinformatic analyses steps that exploit P30 scRNA data^8^ to in part overcome the limitation of bulk gene expression and gain precious information at the cell type level. Based on scRNA, Linnarsson and coworkers defined at least 40 cellular subtypes that constitute the hippocampal tissue of the adult mouse. The integrated analysis of this dataset and a newly generated developmental transcriptome of hippocampus allowed us to infer the individual roles of cell types in the regulation of postnatal synaptogenesis and to predict the differentiation drivers specific to each hippocampal cell class. An extensive literature analysis validated a large part of our predicted drivers, underpinning the effectiveness of our approach. To our knowledge, this is the first comprehensive, entirely data-based prediction of such drivers.

Predictions of the developmental drivers very much depend on the selection of suitable and unambiguous markers from the scRNA data^8^. In fact, for technical and biological reasons, a continuum of intermediate expression levels in between marker genes and unspecifically expressed genes is observable in the data. This complicates the choice of the threshold. Moreover, several genes mark more than one cell (sub)type, which makes their categorization difficult. Nonetheless, we could overcome some of these issues by using a flexible statistical approach for the selection of markers based on an iterative differential expression analysis.

Clearly, our analysis disclosed only a fraction of the whole set of genes implicated in cell differentiation. In fact, we limited our considerations to genes that could be unambiguously identified in adult stages as markers of a specific cell type. In this way, genes which are not expressed anymore at adult stages and/or are multi-functional genes remained undetected in our analysis. An example of multi-functional gene is *Gfap*, which is an established glial cell maker in adult hippocampus that displays a massive postnatal activation in our dataset. However, in the neurogenic niche (sub granular zone) *Gfap* works as a marker of type-1 glia-like stem cell^31^.

On the other hand, the use of adult (P21-P30) markers applied to earlier developmental stages did not seem to cause any bias at the level of cell type assignment, which would arise if a marker switches from one cell type to another during development. While this could be possible, we did not find any such case amongst the validated differentiation drivers (Supplementary Tables S5–S10), suggesting that, exception aside, the general rule for markers is to remain bound to one specific cell type.

In the future research, it will be of particular interest to investigate the features and exact functions of the numerous novel candidates provided in this work. Given its immense importance in the pathophysiology of cognition, the hippocampus is one of the most studied neurodevelopmental models. We now provide a quality dataset of developmental expression data and a plethora of hypotheses that can be used to enhance our mechanistic insights.

## Methods

### RNA-Seq libraries

Embryonic forebrain from E15 mice and hippocampus from P1, P7, P15 and P30 was isolated and snap-frozen in liquid nitrogen. Next, total RNA was extracted using TRIzol (T9424 SIGMA), following the manufacturer’s instructions. Total RNA was treated with DNase (Qiagen, 79254) and purified using an RNeasy MinElute Cleanup Kit (Qiagen, 74204). In total, 2000 ng of total RNA were treated with a Ribo-Zero rRNA Removal Kit (human/mouse/rat; Illumina MRZH11124). The depleted RNA was precipitated for 1 h at −80 °C in three volumes of ethanol plus 1 µg of glycogen. Then, the RNA was washed and resuspended in 36 µl of RNase-free water. RNA fragmentation buffer (NEBNext® Magnesium RNA Fragmentation Module, E6150S) was added to the solution, and the RNA was fragmented by incubation at 95 °C for 3 min. For reverse transcription, cDNA first-strand synthesis was performed with random hexamer primers. cDNA second-strand synthesis was performed with dNTPs to ensure strand specificity. The RNA-Seq library was synthesized using a KAPA Hyper prep kit (KK8504), and a treatment with USER enzyme (NEB, M5505L) was added to digest the non-specific strand. The libraries were pooled (4/lane) on an Illumina HiSeq. 2000. Libraries were sequenced (50 cycles, single-end) yielding on average 40 million mapped reads. RNA-Seq libraries were mapped with GSNAP (version 2015-06-23) against *mm9* mouse RefSeq annotations updated to the 28/7/2015. Quality control (QC) features are shown for each samples in Supplementary Fig. S1E. DESeq 2 (v1.14) was used to perform statistical comparisons.

### Clustering of RNA-Seq time course

After trying a number of different clustering algorithms, including self-organizing map (SOM), WGCNA^32^ and hierarchical clustering with different metrics and linkage functions, we finally opted for a composite system in which an initial trajectory clustering is used to evaluate the optimal number of clusters (trajectories) and, subsequently, clusters are generated via the k-means algorithm.

Initially, pair-wise comparison of gene expression (DESeq 2) were run for all the 10 possible couples of samples amongst E15, P1, P7, P15, P30. Transcripts exceeding DESeq 2 corrected *p-value* = *3e-7* (*Z-score* = *5*) in at least 2 pair-wise comparisons were considered as developmentally regulated. This yielded 13898 transcripts. *Z-scores* resulting from the 10 DESeq 2 pair-wise comparison were assembled in a m*n matrix, where m = 13898 and n = 10 and the matrix was analyzed by “trajectory clustering” that simplifies the values of the input matrix such that each original value is reduced to only 5 possible intervals which are delimited by the 10^th^, 30^th^, 70^th^ and 90^th^ percentiles. These 5 values can be generally interpreted as “not changed”, “up(down)-regulated”, “strongly up(down)-regulated” or, equally, “medium”, “above(below)-average”, “strongly above(below)-average”. Subsequently, each element is associated with a trajectory (a unique tuple of these 5 values) and the trajectories collecting enough elements are recognized as the real/main trajectories. The minimal number of elements that a real trajectory must collect is set with the parameter *min_el*. Here, we used very low *min_el* in order to retain as many trajectories as possible, to minimize loss of information. More precisely, *min_el was* set to *1%*, meaning that a trajectory is considered as real if it collects at least *1%* of the total elements to be clustered. The final number of real trajectories is equivalent to the optimal number of clusters. Once determined the optimal amount of clusters, the m*n matrix (with its original values) is then clustered via standard k-means with squared Euclidean distance. In our case, the optimal number of clusters was set to 13. The m*n matrix was then clustered in 13 groups via standard k-means clustering with Euclidean distance that proved to be the best among several different linkages and distance metrics.

### Enrichments

Standard hypergeometric tests with *Benjamini* (GO enrichments) or *Bonferroni* (other enrichments) correction were used to determine the enrichment in MATLAB environment. GO annotations are updated to 25/6/2015 and GSEA annotations to 20/10/2015.

### Analysis of single cell RNA-Seq data

Data from^8^ was analyzed in order to extract the list of markers for each cell types and subtypes. As previously done in^8^ all the cells coming from P21-P30 mice were used together, as no age effect of cell phenotypes was found. In the supplementary dataset containing cell type identities (http://linnarssonlab.org/cortex/), 7 main cell types are presented, whereas the main manuscript^8^ describes 9 main cell types. This is because in the supplementary dataset astrocytes and ependymal cells are pooled in one population and endothelial and mural cells are pooled in another population. Similarly, also perivascular macrophages and microglia ware pooled in one population and choroid cells and astrocytes in another. We decided not to pool cell types with different identities, phenotypes and names into larger groups, so we considered perivascular macrophages and choroid cells as separate cell types and not subtypes, resulting in 11 total cell types and 47 cell subtypes. As a first step, we run 47*46/2 = 1081 pair-wise comparisons of expression (DESeq 2) among the 47 described cellular subtypes. The markers of each cell subtype were defined as those significantly up-regulated (DESeq 2 corrected *p-value* < *0*.05) in at least 44/46 cases. We did not ask 46/46 because several genes are “multiple” markers, as they are expressed in two or three different cell subtypes. Setting 46/46 cases would then cause their loss and a consequent loss of information. These multiple markers were consequently retained, assigned to the cell subtype of highest expression. An analogous method was used to calculate the markers for the 11 major cell types. Essentially, genes were considered as markers of a whole cell type when i) they were homogeneously expressed (DESeq 2 corrected *p-value* > 0.05) within the cells of a given cell type ii) they are up-regulated (DESeq 2 corrected *p-value* < 0.05) in comparison to cells belonging to a different cell type.

### Live vertebrates regulations

All experiments on animals were carried out in accordance with the approved animal care and use guidelines of the Animal Care Committee, Radboud University Nijmegen Medical Centre (RU-DEC-2011-021, protocol number: 77073).

### Data Access

Data deposited in GEO, ID GSE79380.

MATLAB scripts are available at: https://github.com/iaconogi/Trajectory-clustering.

## Electronic supplementary material

Supplementary Information
