# Supplementary material for: Integrated transcriptional analysis unveils the dynamics of cellular differentiation in the developing mouse hippocampus
Source: Sci Rep. 2017 Dec 22;7:18073. doi: 10.1038/s41598-017-18287-w (PMC5741714; doi:10.1038/s41598-017-18287-w)
Supplement: Supplementary file 1 — Supplementary Information [file 41598_2017_18287_MOESM1_ESM.pdf]

SUPPLEMENTARY NOTES

Integrated transcriptional analysis unveils the dynamics of cellular differentiation in the developing mouse hippocampus

Giovanni Iacono[1,*], Marco Benevento[2,3,4], Aline Dubos[5], Yann Herault[5], Hans van Bokhoven[2,3,4], Nael Nadif Kasri[2,3,4] and Hendrik G. Stunnenberg[1,*].

[1] Radboud University, Department of Molecular Biology, Faculty of Science, 6500 HB Nijmegen, the Netherlands

[2] Department of Cognitive Neuroscience, Radboudumc, 6500 HB Nijmegen, the Netherlands

[3] Department of Human Genetics, Radboudumc, 6500 HB Nijmegen, the Netherlands

[4] Donders Institute for Brain, Cognition, and Behaviour, Centre for Neuroscience, 6525 AJ Nijmegen, the Netherlands

[5] Institut Clinique de la Souris, PHENOMIN, GIE CERBM, 1 rue Laurent Fries, 67404 Illkirch, France

*Corresponding Authors: H.Stunnenberg@ncmls.ru.nl, giovanni.iacono@cnag.crg.eu

Complementary notes on the predicted drivers

Together with validated differentiation drivers, many novel candidates emerged from the data. Among the interneurons predicted drivers, *Arl4c* represents a compelling example. *Arl4c* belongs to the family of three Arf-like GTPases, namely *Arl4a*, *Arl4c*, and *Arl4d*, that were shown in *HeLa* cells to be implicated in the cytohesins-mediated activation of *Arf6*, which in turn regulates endocytosis, actin dynamics, and cell adhesion [1]. However, for these proteins nothing is known in the context of the CNS. Our integrated analysis indicates that *Arl4c* is the only member of the family expressed in interneurons and that its expression significantly drops after the 1st postnatal week. This indicates that *Arl4c* may be implicated in the development of neonatal interneuronal circuitry, possibly by the mediation of a change in the GTP-signaling. In the case of pyramidal neurons, most of our predicted drivers were already directly linked to neurogenesis, such as *Foxp1* [2], while some others have been believed to be linked to neurogenesis for a long time but still lack clear information about their exact mechanisms. One such example is *Nnat*, which was firstly observed as regulated in brain development more than 20 years ago ([3], 1994). For oligodendrocytes and astrocytes the situation is different, as the largest majority of candidates results to be novel. An interesting example is the gene *Nkain1*, a protein interacting with the Na+/K+ Transporting ATPase, which was initially described in Drosphila [4] and was also identified by genome-wide association as a significant risk factor for alcohol dependence [5]. Overall, the clues about the roles of *Nkain1* in the CNS are very scarce and this gene was never directly linked to oligodendrocytes. In our dataset, *Nkain1* expression is high in the embryonic forebrain and after that progressively decreases and reaches its minimum at the beginning of the synaptogenesis phase (around the 2nd postnatal week), which clearly indicates that it has a role in the perinatal stages of hippocampus development, possibly by a modulation of oligodendrocytes differentiation or behavior. Intriguingly, in the set of oligodendrocytes predicted drivers we also found the histone coding proteins *Hist1h2bc* and *Hist1h4h*; many studies have confirmed aberrant expression of several histone proteins in bipolar disorders and schizophrenia and here we provide a clear link between a number of them and hippocampus development. In the case of ependymal cells, the situation is slightly different because their markers are prone to follow a peculiar decreasing pattern during development, thus depriving C1-C5 from being the only minor representatives of decreasing genes as it was for the other neuronal and glial cells. Nevertheless, in C1-C5 we could find the genes *Cd24a*, *Pdpn* and *Socs3*, which have proved to be involved in ependymal cells survival and differentiation (Tables S5-S11). Other ependymal genes in C1-C5 were previously linked to neurogenesis and development but not recognized as ependymal markers, such as the transmembrane receptor *Plxnb2* (Tables S5-S11). A number of our predicted ependymal-specific drivers of differentiation were previously linked to developmental disorders, such as *Tspan6* (intellectual disability[6]) and *Tuba1a* (encephalic malformations[7]) or tumorigenesis, such as *Nuf2*, a component of a protein complex associated with the centromere whose knockout caused the suppression of glioma growth [8]. *Nuf2* physiological expression is high in perinatal stages and subsequently drops of approximately 10-fold after in the course of the 1st postnatal week. Once

again, as it was for astrocytes and oligodendrocytes, aberrant expression of developmentally-expressed genes causes tumorigenesis in adult tissue. Also in the case of the minor populations of cells, perivascular macrophages and mural cells, we could identify a number of genes expressed predominantly at earlier developmental stages. However, these cell types do not exhibit the strong disparity in enrichments seen in the other main cell-types and therefore we cannot conclude that their early markers are candidates for driving differentiation. However, these genes likely play crucial role in the developmental program and are of particular interest because they belong to the cell-types that are usually not considered in hippocampus.

Finally, in just one case a predicted driver appeared in two different cluster. In fact, *Bcl11a*, which was previously involved in axon branching and dendrite outgrowth [9], presents one transcript variant (*NM_001242934*) which is clustered in C2 and displays a by a peak of expression in P1 , while the other isoforms all belong to C1 (peak in embryonic stage). This may imply a specific role for *NM_001242934* in the first postnatal days.

SUPPLEMENTARY FIGURE LEGEND

Supplementary Figure 1. (a,b) GO comparisons, Radar plots in which outer rings correspond to higher significance. Each radar plot includes the two compared clusters plus a third shape (gray) that depicts the highest enrichment found in the remaining 11 clusters. This layout easily visualizes the differences between the analyzed clusters and, at the same time, quantifies the overall specificity of their enriched GO terms. C1 and C4 show distinct enrichments in the gene ontology: patterning and regionalization genes are markedly repressed in the transition from embryo to P1 (C1) while proliferation/differentiation genes are more gradually de-activated throughout the whole postnatal development (C4). Similarly, also C6 and C9 show non overlapping enrichments. For instance, the genes implicated in the hexose metabolism are activated at later stages (C9) as opposed to synaptogenesis genes (C6). (**c**) Distinct pathways for developmental and adult neurogenesis. GO enrichments (y-axis, *Benjamini* corrected *p-values* in *Z-score* scale) delineate the numerous unique functions and pathways, as well as the common properties, that mark developmental and adult neurogenic genes. (**d**) Developmental change in the subunit composition of the Kv3 potassium channels. While the genes of the subunits kv3.1, kv3.3 and kv3.4 reach the peak of expression at P30, the subunit kv3.2 (gene KCNC2, mean +/- SEM) is expressed earlier in development and reaches the peak already at P15. (**e**) Mapping statistics for RNA-seq samples.

All the Supplementary tables can be downloaded from GEO (GSE79380)

TableS1.xlsx: Complete lists of genes and transcripts of the 13 developmental clusters.

TableS2.xlsx: GO enrichments of the 13 developmental clusters. *Bonferroni* corrected *p-values* are represented in *Z-score* values.

TableS3.xlsx: Lists of markers of cell types and cell subtypes.

TableS4.xlsx: Developmental profiles of markers.

TableS5.xlsx: Validation of astrocytes drivers

TableS6.xlsx: Validation of ependymal drivers

TableS7.xlsx: Validation of interneurons drivers

TableS8.xlsx: Validation of macrophages drivers

TableS9.xlsx: Validation of oligodendrocytes drivers

TableS10.xlsx: Validation of pyramidal neurons drivers

References

[1] I. Hofmann, A. Thompson, C. M. Sanderson, and S. Munro, "The arl4 family of small g proteins can recruit the cytohesin arf6 exchange factors to the plasma membrane." *Current biology : CB*, vol. 17, no. 8, pp. 711–716, Apr 2007. [Online]. Available: http://www.ncbi.nlm.nih.gov/pubmed/17398095

[2] C. Bacon, M. Schneider, C. Le Magueresse, H. Froehlich, C. Sticht, C. Gluch, H. Monyer, and G. A. Rappold, "Brain-specific foxp1 deletion impairs neuronal development and causes autistic-like behaviour." *Molecular psychiatry*, vol. 20, no. 5, pp. 632–639, May 2015. [Online]. Available: http://www.ncbi.nlm.nih.gov/pubmed/25266127

[3] R. Joseph, D. Dou, and W. Tsang, "Molecular cloning of a novel mrna (neuronatin) that is highly expressed in neonatal mammalian brain." *Biochemical and biophysical research communications*, vol. 201, no. 3, pp. 1227–1234, Jun 1994. [Online]. Available: http://www.ncbi.nlm.nih.gov/pubmed/8024565

[4] S. Gorokhova, S. Bibert, K. Geering, and N. Heintz, "A novel family of transmembrane proteins interacting with beta subunits of the na,k-atpase." *Human molecular genetics*, vol. 16, no. 20, pp. 2394–2410, Oct 2007. [Online]. Available: http://www.ncbi.nlm.nih.gov/pubmed/17606467

[5] L. Zuo, K. Wang, X.-Y. Zhang, J. H. Krystal, C.-S. R. Li, F. Zhang, H. Zhang, and X. Luo, "Nkain1-serinc2 is a functional, replicable and genome-wide significant risk gene region specific for alcohol dependence in subjects of european descent." *Drug and alcohol dependence*, vol. 129, no. 3, pp. 254–264, May 2013. [Online]. Available: http://-www.ncbi.nlm.nih.gov/pubmed/23455491

[6] A. K. Vincent, A. Noor, A. Janson, B. A. Minassian, M. Ayub, J. B. Vincent, and C. F. Morel, “Identification of genomic deletions spanning the pcdh19 gene in two unrelated girls with intellectual disability and seizures.” *Clinical genetics*, vol. 82, no. 6, pp. 540–545, Dec 2012. [Online]. Available: http://www.ncbi.nlm.nih.gov/pubmed/22091964

[7] S. Yokoi, N. Ishihara, F. Miya, M. Tsutsumi, I. Yanagihara, N. Fujita, H. Yamamoto, M. Kato, N. Okamoto, T. Tsunoda, M. Yamasaki, Y. Kanemura, K. Kosaki, S. Kojima, S. Saitoh, H. Kurahashi, and J. Natsume, “Tuba1a mutation can cause a hydranencephaly-like severe form of cortical dysgenesis.” *Scientific reports*, vol. 5, p. 15165, Oct 2015. [Online]. Available: http://www.ncbi.nlm.nih.gov/pubmed/26493046

[8] S.-K. Huang, J.-X. Qian, B.-Q. Yuan, Y.-Y. Lin, Z.-X. Ye, and S.-S. Huang, “Sirna-mediated knockdown against nuf2 suppresses tumor growth and induces cell apoptosis in human glioma cells.” *Cellular and molecular biology (Noisy-le-Grand, France)*, vol. 60, no. 4, pp. 30–36, Nov 2014. [Online]. Available: http://www.ncbi.nlm.nih.gov/pubmed/-25481014

[9] T.-Y. Kuo, C.-Y. Chen, and Y.-P. Hsueh, “Bcl11a/ctip1 mediates the effect of the glutamate receptor on axon branching and dendrite outgrowth.” *Journal of neurochemistry*, vol. 114, no. 5, pp. 1381–1392, Sep 2010. [Online]. Available: http://www.ncbi.nlm.nih.gov/pubmed/20534004

SUPPLEMENTARY FIGURE 1

a

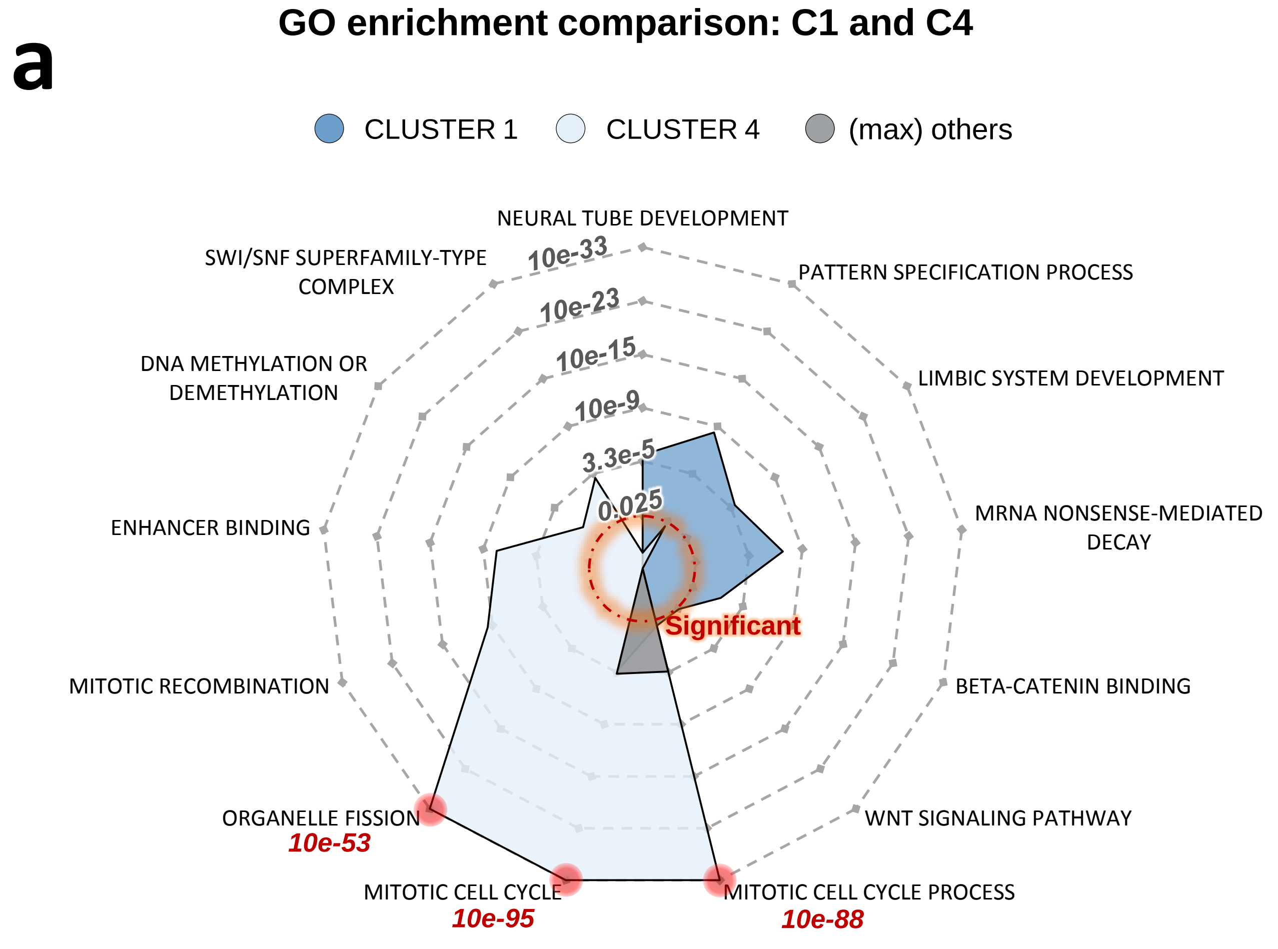

b

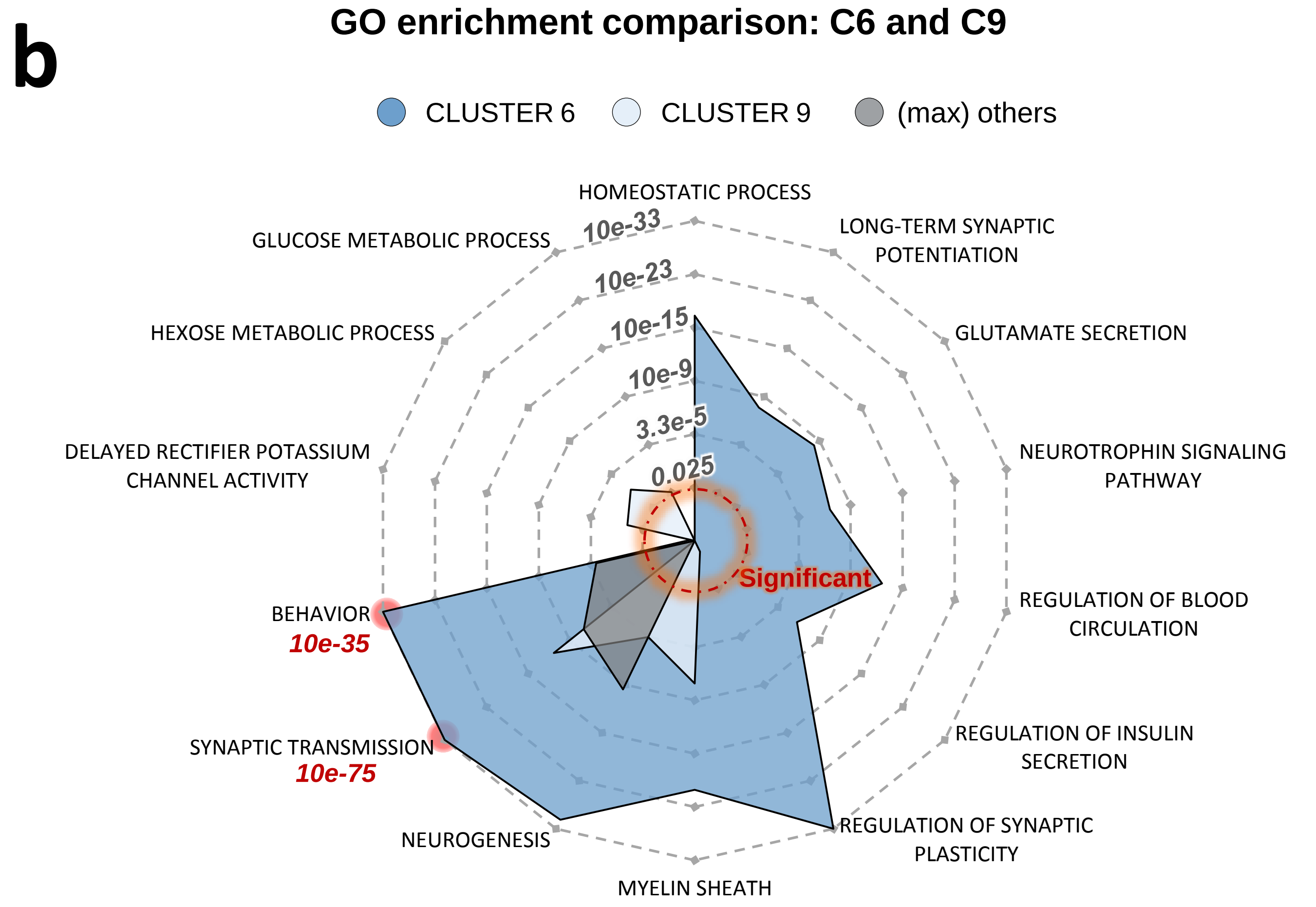

c

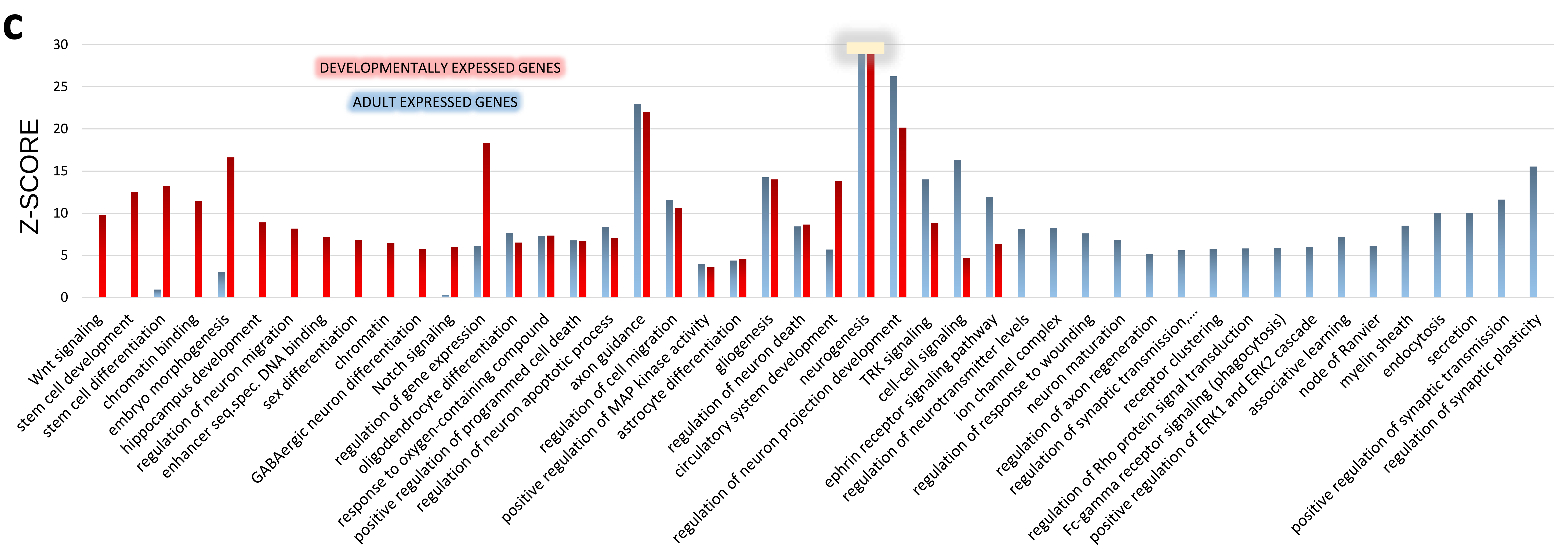

d

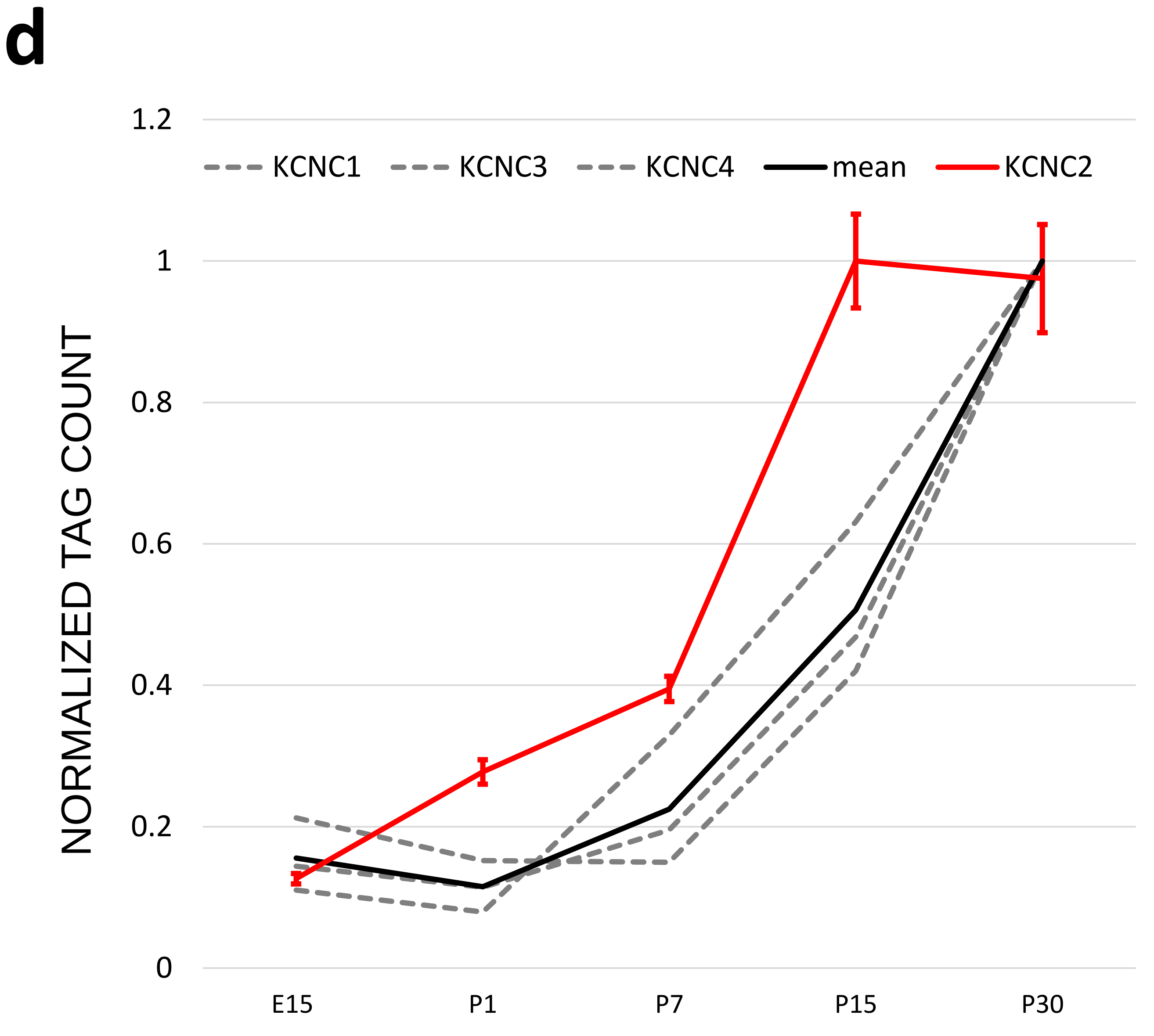

e

	E15_rep1	E15_rep2	E15_rep3
Total mapped reads	42,769,847	45,527,151	47,536,400
Mapped forward strand	48%	48%	47%
Mapped reverse strand	52%	52%	53%
Mapped to repeats	734,163	941,504	1,027,063
Mapped to repeats (%)	1.7%	2.1%	2.2%

	P1_rep1	P1_rep2	P1_rep3	P1_rep4
Total mapped reads	35,454,537	35,434,228	34,937,102	30,089,333
Mapped forward strand	50.66%	52.59%	50.78%	50.77%
Mapped reverse strand	49.34%	47.41%	49.22%	49.23%
Mapped to repeats	844,517	803,704	860,917	656,101
Mapped to repeats (%)	2%	2%	2%	2%

	P7_rep1	P7_rep2	P7_rep3
Total mapped reads	29,367,832	25,037,768	23,791,820
Mapped forward strand	50.62%	50.85%	50.70%
Mapped reverse strand	49.38%	49.15%	49.30%
Mapped to repeats	603,859	492,533	510,580
Mapped to repeats (%)	2.06%	1.97%	2.15%

	P15_rep1	P15_rep2	P15_rep3
Total mapped reads	31,671,695	29,270,453	31,961,647
Mapped forward strand	50%	50%	50%
Mapped reverse strand	50%	50%	50%
Mapped to repeats	562,789	570,952	637,673
Mapped to repeats (%)	1.8%	2.0%	2.0%

	P30_rep1	P30_rep2	P30_rep3	P30_rep4	P30_rep5	P30_rep6
Total mapped reads	46,940,501	32,737,488	45,198,494	44,629,410	46,054,557	98,744,472
Mapped forward strand	41.90%	50.29%	41.57%	42.02%	39.60%	42.12%
Mapped reverse strand	58.10%	49.71%	58.43%	57.98%	60.40%	57.88%
Mapped to repeats	771,709	556,775	4,298,292	4,536,334	3,112,229	4,521,536
Mapped to repeats (%)	1.64%	1.70%	9.51%	10.16%	6.76%	4.58%
